# Supplementary material for: Causal Relationship between Aging and Anorexia Nervosa: A White-Matter-Microstructure-Mediated Mendelian Randomization Analysis
Source: Biomedicines. 2024 Aug 16;12(8):1874. doi: 10.3390/biomedicines12081874 (PMC11351342; doi:10.3390/biomedicines12081874)
Supplement: Supplementary file 1 [file biomedicines-12-01874-s001.zip › Supplementary Figure/Figure S8/Leaveoneout plot of MR analysis for TL to ubm-b-1479.pdf]

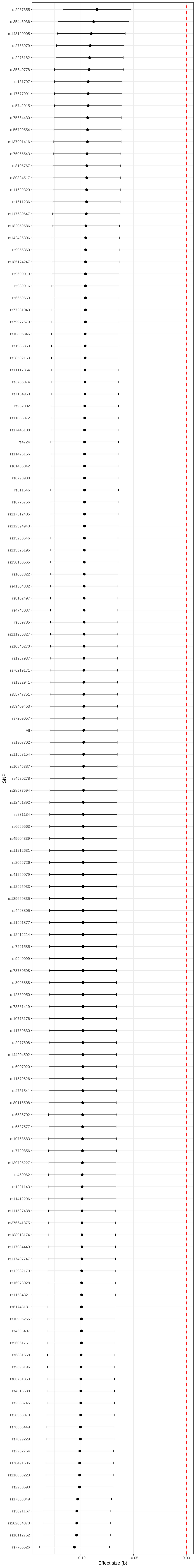
rs2967355
rs35446936
rs143190905
rs2763979
rs2276182
rs35640778
rs131797
rs17677991
rs5742915
rs75664430
rs56799554
rs137901416
rs76065543
rs8105767
rs80324517
rs11699829
rs1611236
rs117630647
rs182059586
rs142426306
rs9953360
rs185174247
rs9600019
rs939916
rs6659669
rs77231040
rs79977579
rs10805346
rs1985369
rs28502153
rs11117354
rs3785074
rs7164950
rs932002
rs11085072
rs17445108
rs4724
rs11426156
rs61405042
rs6790988
rs611646
rs6776756
rs117512405
rs112394943
rs13230646
rs113525195
rs150150565
rs1003322
rs41304832
rs8102497
rs4743037
rs869785
rs111950327
rs10840270
rs1957937
rs76219171
rs1332941
rs55747751
rs59409453
rs7209057
All
rs1907702
rs11557154
rs10845387
rs4530278
rs28577594
rs12451892
rs871134
rs6669563
rs45604339
rs11212631
rs2056726
rs41269079
rs12925933
rs139669835
rs4498805
rs11991877
rs12412214
rs7221585
rs9940099
rs73730598
rs3093888
rs12369950
rs73581419
rs10773176
rs11769630
rs2977608
rs144204502
rs6007020
rs11579626
rs4731541
rs80116508
rs6536702
rs6587577
rs10768683
rs7790856
rs139795227
rs450962
rs1291143
rs11412296
rs111527438
rs376641875
rs188918174
rs117034449
rs117407747
rs12932179
rs16978028
rs11584821
rs61748181
rs10905255
rs4695407
rs56061761
rs6881568
rs9398196
rs66731853
rs4616688
rs2538745
rs28363070
rs76666449
rs7099229
rs2282784
rs78491606
rs116863223
rs2230590
rs17803849
rs3891167
rs202034370
rs10112752
rs7705526
SNP
-0.10
-0.05
0.00
Effect size (b)
